# Insecticidal Activity of *Hyoscyamus niger* L. on *Lucilia sericata* Causing Myiasis

**DOI:** 10.3390/plants9050655

**Published:** 2020-05-22

**Authors:** Esra Küpeli Akkol, Mert Ilhan, Esma Kozan, Fatma Tuğçe Gürağaç Dereli, Mustafa Sak, Eduardo Sobarzo-Sánchez

**Affiliations:** 1 Department of Pharmacognosy, Faculty of Pharmacy, Gazi University, Etiler, Ankara 06330, Turkey; banotu@hotmail.com; 2 Department of Pharmacognosy, Faculty of Pharmacy, Van Yüzüncü Yıl University, Tuşba, Van 65080, Turkey; mertilhan@yyu.edu.tr; 3 Department of Parasitology, Faculty of Veterinary Medicine, Afyon Kocatepe University, Afyonkarahisar 03200, Turkey; esmakozan@aku.edu.tr; 4 Department of Pharmacognosy, Faculty of Pharmacy, Suleyman Demirel University, Isparta 32260, Turkey; tugcedereli@sdu.edu.tr; 5 Instituto de Investigación e Innovación en Salud, Facultad de Ciencias de la Salud, Universidad Central de Chile, Santiago 8330507, Chile; eduardo.sobarzo@ucentral.cl or; 6 Department of Organic Chemistry, Faculty of Pharmacy, University of Santiago de Compostela, 15782 Santiago de Compostela, Spain

**Keywords:** black henbane, *Hyoscyamus niger*, *Lucilia sericata*, myiasis, Solanaceae

## Abstract

Background: *Hyoscyamus niger* L. (Solanaceae) generally known as henbane, is commonly distributed in Europe and Asia. In Turkey, henbane seeds have been used in folk medicine to remove worms from the eyes. The present study aimed to investigate the insecticidal activity of *H. niger* seeds. Methods: *n*-hexane, ethyl acetate, methanol and alkaloid extracts were prepared from the seeds of the plant and their insecticidal activities on *Lucilia sericata* larvae were evaluated. EC_50_ and EC_90_ values of the alkaloid extract were calculated and morphological abnormalities were investigated. Results: Alkaloid extract prepared from the seeds of this plant displayed significant insecticidal activity. EC_50_ values of *H. niger* seeds alkaloid extract were found to be 8.04, 8.49, 7.96 μg/mL against first, second and third instar, respectively. It was determined that malformations of larvae included damaged larvae with small size, contraction and weak cuticle. Furthermore, HPLC analysis was performed on alkaloid extract of *H. niger* seeds and main components of the extract were determined. It was determined that alkaloid extract mainly contain hyoscyamine and scopolamine. Conclusions: These results confirm the folkloric usage of the plant and suggest that the alkaloid content of the plant could be responsible for the insecticidal activity.

## 1. Introduction

*Hyoscyamus* genus has six species in the flora of Turkey, *H. aureus*, *H. albus*, *H. leptoclyx*, *H. niger*, *H. pusillus* and *H. reticulatus* [1]. The genus belongs to Solanaceae family. *Hyoscyamus niger* L. (black henbane) is the most popular species of *Hyoscyamus* genus. It has been used as a medicinal plant since ancient Greece [2]. The mature corolla of *H. niger* is lurid yellow, usually veined purple; the fruiting calyx is constricted at the middle; and the upper cauline leaves are amplexicaul [1]. *H. niger* leaves are used as an antispasmodic for overfed animals and the seeds are used for itching, reddening in eyes, and earache [3,4]. One of the most popular uses of *H. niger* in folk medicine is to expel worms in the mouth or eyes [3,5,6,7,8,9]. Seeds are spread on dying embers and covered with a blanket. Eyes of the patient are exposed to the vapor under the blanket. After the vapor application, small white worms with black heads drop from the eyes or mouth [5,7,9,10]. According to phytochemical studies, all parts of the plant contain hyoscyamine and scopolamine [2,11]. In addition to these compounds, four lignanamides, a tyramine derivative, and ten other nonalkaloidal components were isolated from the seeds of *H. niger.* Among them, hyoscyamide, 1,24-tetracosanediol diferulate, and 1-*O*-(9Z,12Z-octadecadienoyl)-3-*O*-nonadecanoyl glycerol are new structures. The other compounds were identified as grossamide, cannabisin D, cannabisin G, *N*-trans-feruloyl tyramine, 1-*O*-octadecanoyl glycerol, 1-*O*-(9Z,12Z-octadecadienoyl) glycerol, 1-*O*-(9Z,12Z-octadecadienoyl)-2-*O*-(9Z,12Z-octadecadienoyl) glycerol, 1-*O*-(9Z,12Z-octadecadienoyl)-3-*O*-(9Z-octadecenoyl) glycerol, rutin, vanillic acid, β-sitosterol, and daucosterol [12]. Furthermore, previous studies reported that *H. niger* seeds displayed an inhibitory effect some pathogens including six *Candida* species, *Enterococcus faecalis, Escherichia coli, Klebsiella pneumoniae, Pseudomonas aeruginosa* and *Proteus mirabilis* [13,14]. 
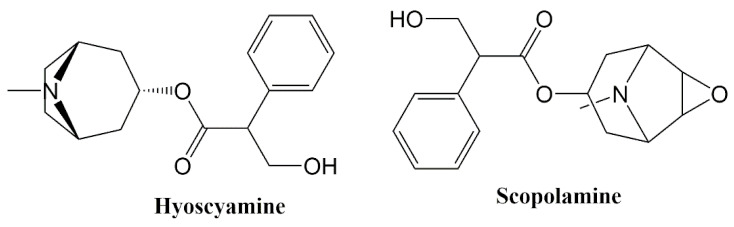


*Lucilia sericata* (Meigen) (Diptera: Calliphoridae) is a facultative ectoparasite used for necrotic wounds that conventional drugs can not treat [15] and is commonly known as the sheep blow fly or the green bottle fly. However, it can give rise to myiasis in humans. Myiasis is the invasion of animal and human tissues and organs by the larval stages of dipterous flies [16] causing either facultative or obligatory myiasis [17]. Myiasis usually occurs in regions, where people encounter animals [18]. Most patients live in economically disadvantaged, overcrowded, fly-infested areas [19]. Nosocomial myiasis in hospitalized patients happens with moderate incidence. Confined to bed patients with open wounds might have infestations if *L. sericata* are present [20,21].

In the light of these facts, this study evaluated the antilucidal activity of four extracts obtained from *H. niger* seeds, growing wild in Turkey and also HPLC analysis were conducted to identify the major components in the alkaloid extract of *H. niger* seeds which showed the highest inhibitory activity against *L. sericata*.

## 2. Results

The results indicated that while the alkaloid extract of *H. niger* showed high activity, the methanol extract showed moderate activity on the development of first instar of *L. sericata*. As the concentration increased, the *H. niger* alkaloid extract insecticidal effect increased. 100% larva mortality was reached after application of 32 μg/mL alkaloid extract. Larva mortality reached 60% and 81% at concentrations of 8 and 16 μg/mL, respectively (Table 1).

The insecticidal activity of *H. niger* alkaloid extract was considerably active on the development of *L. sericata* second instar. The mortality rate was 100% at 32 μg/mL and 89% at 16 μg/mL in the alkaloid extract group. The mortality rate was 52% at 32 μg/mL in the methanol group (Table 2). The 16 and 32 μg/mL concentrations of the alkaloid extract induced 100% mortality for the third instar (Table 3).

**Figure 1:**
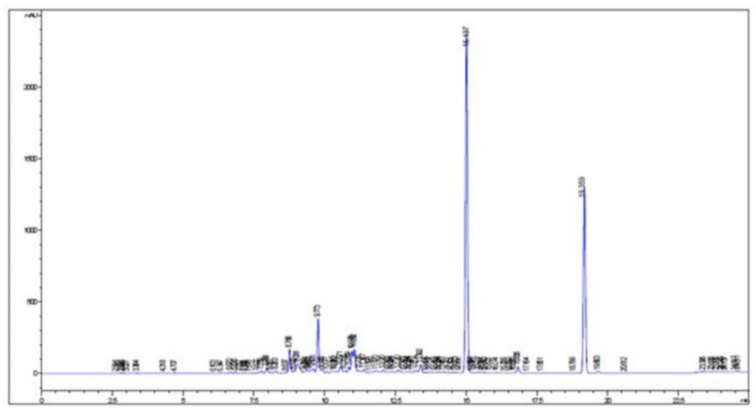
HPLC chromatogram of the alkaloid extract obtained from *H. niger* seeds.

According to the HPLC studies, the amount of scopolamine and hyoscyamine were determined as 0.1578 (*g/g*) and 0.1256 (*g/g*), respectively (Figure 1).

For the alkaloid extract, EC_50_ and EC_90_ values were calculated. According to the results, EC_50_ values of *H. niger* alkaloid extract were found to be 8.04, 8.49, and 7.96 μg/mL against first, second, and third instar, respectively. On the other hand, EC_90_ values of *H. niger* alkaloid extract were found to be 30.95, 17.79, and 12.35 μg/mL against first, second, and third instar, respectively.

## 3. Discussion

The current study describes the effects of the extracts prepared from *H. niger* seeds on *L. sericata* larvae which causes myiasis in humans. Myiasis is explained as the invasion of living vertebrates (animals and/or humans) by dipterous larvae.

In humans, dipterous larvae may feed on the host’s alive or dead tissue as well as liquid body substances and give rise to broad infestations liable on the body location [22]. Wound myiasis results from flies of Calliphoridae which includes *L. sericata*. It can cause ocular myiasis and nasal myiasis [23,24,25]. There are three main methods for the treatment of myiasis: (I) the use of a toxic substance for larvae, (II) the production of confined hypoxia to power the appearance of the larva, and (III) the mechanical or medical elimination of the maggots [22]. *H. niger* seeds have been used to remove worms from eyes in folk medicine [5,7,9,10]. The presence of insecticidal activities of the oils obtained from *Apium graveolens*, *Brassica compestris, Raphanus sativus* and *Trigonella foenum-graecum*, was reported by Khater and Khater [21]. Their results suggested that oils obtained from those four plants might signify novel and safe possible insecticides for the control of blowflies. In the light of these facts, our study investigated the effects of *H. niger* seeds on *L. sericata* which cause myiasis. Behravan et al. [26] reported that *H. niger* flower extract could be used to fight *Anopheles* spp mosquito larvae. They found that the most active extract for destroying the mosquitoes *Anopheles* spp larvae was the henbane flower. Furthermore, Wang et al. [27] exhibited that the ethanol extract of *H. niger* seeds showed high insecticidal activity against the *Aphis laburni* Kaltenbach. After the alkaloid extract of *H. niger* seeds, morphological malformation of larvae involved contractile, small sized, and damaged larvae with weak cuticles. Similar results have been described following the treatment of anise, chamomile and rosemary oils as well lettuce against larvae of *L. sericata* [28]. These abnormalities also have been seen in *Chrysomya albiceps* which causes myiasis with the treatment of *Punica granatum* [29]. Moreover, *Allium cepa*, *Nigella sativa* and *Sesamum indicum* oils seriously affect pupation rates and appearance of adult *Culex pipiens* and *Musca domestica* [30]. Three endocrine glands are responsible for releasing neuro-hormones vital for growth, development and differentiation; the corpus cardiacum, corpus allatum, and prothoracic gland in larva insects. It has been presented that plant components cause advanced degeneration of all these endocrine glands in larvae [31]. This morphological degeneration indicates a comprehensive dysfunction of the neuroendocrine system.

Black henbane contains alkaloids such as hyoscyamine, atropine, tropane and scopolamine. Chromatographic analysis of the alkaloid percentages from *H. niger* identified in the leaves, roots and seeds are 0.17, 0.08 and 0.05, respectively [32,33]. Ghorbanpour et al. [2] found that the leaves of *H. niger* include hyoscyamine and scopolamine in the yield of 0.725 and 0.362 (*g/g* plant), respectively, and Ma et al. [12] isolated nonalkaloid compounds from *H. niger* seeds. On the other hand, our results exhibited that the amount of scopolamine and hyoscyamine were determined as 0.1578 (*g/g*) and 0.1256 (*g/g*), respectively, in the seeds of *H. niger*. Tropane alkaloids can induce antispasmodic effects of smooth muscle, reduction of bronchial hypersecretions, and relief of gastric pain [34]. In addition to alkaloids, henbane seeds contain withanolides, flavonoids, lignans, coumarinolignans, saponins, glycerides, glycosides and phenolics [32]. Begum [32] found that because of its nonalkaloidal constituents, henbane seeds possess antimicrobial, antidiarrheal, antispasmodic, anticonvulsant, anti-inflammatory, analgesic and antipyretic activities. Swathi et al. [35] reported that *Datura stramonium,* which belongs to Solanaceae family, has larvicidal and mosquito repellent activities. However, hyoscyamine and scopolamine possess central effects of anticholinergic toxicity including confusion, delirium, irritability, agitation, hallucinations (typically visual and/or tactile), seizures and mydriasis [36]. Hyoscyamine and scopolamine are major compounds of this plant. Therefore, the effects of *H. niger* seeds against *L. sericata* could be due to their hyoscyamine and scopolamine.

## 4. Materials and Methods

### 4.1. Plant Material

The plant, *H. niger*, was collected from Ankara in June 2015 and authenticated by Prof. Dr. Murat EKİCİ from Gazi University, Faculty of Science and Art, Department of Biology, Ankara. A voucher specimen (ANK10016) was deposited in the Ankara University, Herbarium of Faculty of Science, Ankara, Turkey.

### 4.2. Preparation of n-Hexane, Ethyl Acetate and Methanol Extracts

Plant material (500 g) was dried in the shade and the seeds were extracted with *n*-hexane [5 L], ethyl acetate [5 L] and methanol [5 L] successively at room temperature for 48 h (5 × 4 L). Extracts were vaporized under reduced pressure at 40 °C. The yields of each extract were 10.94% [54.7 g] for *n*-hexane, 16.22% [81.1 g] for ethyl acetate, and 37.13% [185.65 g] for methanol extract.

### 4.3. Preparation of Alkaloid Extract

For the extraction of the alkaloid extract, 10 L CH_2_CI_2_-MeOH-NH_4_OH (15:5:1) was added to *H. niger* seeds (100 g), sonicated for 10 min, then saved at room temperature for 1 h. Following filtration, the residue was washed twice with CH_2_CI_2_ (1 L). The filtrate was evaporated to dryness. Five liters of CH_2_CI_2_ and 2 L of 1 N H_2_SO_4_ were added to the residue, then the solution was mixed. The CH_2_CI_2_ phase was removed and the H_2_SO_4_ phase was attuned to pH 10 with 28% NH_4_OH. Finally, alkaloids were extracted with once 2 L and twice with 1 L of CH_2_CI_2_. The collective extracts were filtered after adding anhydrous Na_2_SO_4_ and then the residue was washed using 1 L of CH_2_CI_2_. The combined filtrates were vaporized to dryness at 40 °C [37]. The yield of the alkaloid extract was 5.28% [5.3 g].

### 4.4. Obtaining First, Second and Third Instars

**Figure 2:**
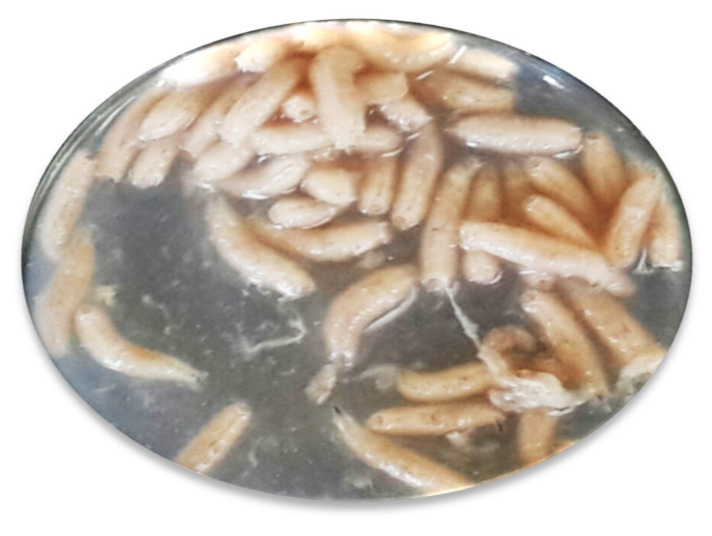
Third instars larvae of *Lucilia sericata* obtained under laboratory conditions. The larvae showed the typical maggot body shape.

The adult *L. sericata* flies were collected from the Tazlar village of Afyonkarahisar province using fly netting. Flies were reared in the laboratory according to El-Khateeb et al. [38]. Beef was used as bait for collecting flies in open area. In the laboratory, first stage larvae were obtained 8–12 h after hatching, according to temperature. Second stage larvae were obtained after 31 h and third stage larvae after 72 h according to temperature and humidity (Figure 2) [39].

### 4.5. Mounting of L. sericata Larvae

For comprehensive morphological studies of normal larvae, the larvae were washed more than a few times with saline, placed in 5% caustic soda (NaOH) and incubated at room temperature for 1–2 h for the first instar or overnight for the second and third instar. The larvae were evacuated from their contents, washed with water, and dehydrated through ascending serial concentrations of ethanol (70, 80, 90 and 100%) for 1 h each. Lastly, they were cleared in clove oil and washed in xylene. The larvae were mounted in Canada balsam and incubated in an oven at 40 °C to dry for 24 h [40].

### 4.6. Identification of L. sericata Larvae and Adult

**Figure 3:**
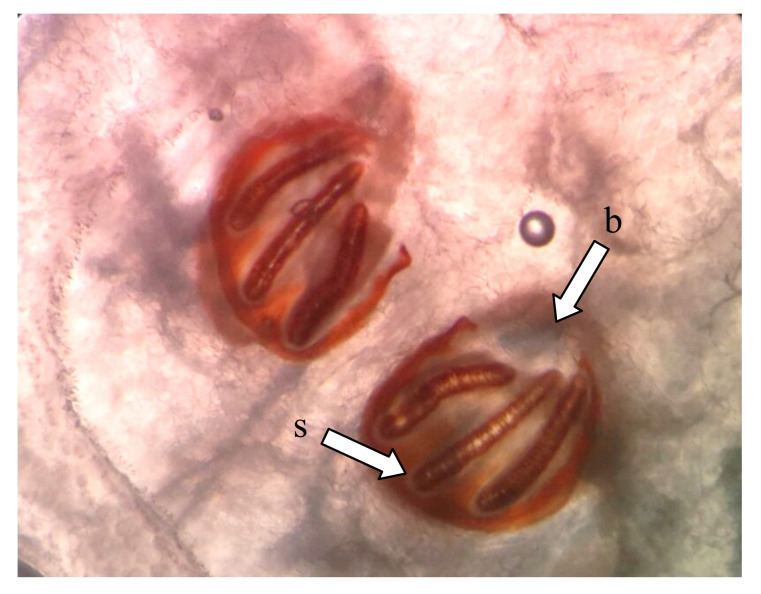
Light microscopic view of the posterior part of the maggot of *Lucilia sericata*. The button (b) presented on both spiracles and the three slits (s) in each posterior spiracle were straight.

The identification was approved according to Holloway [41] and Zumpt [16]. Examination of posterior spiracles on a glass slide under light microscope (Olympus CX-21, Japan) showed two *L. sericata* (Figure 3).

### 4.7. Ingestion Assay

Early first, second and third instars of *L. sericata* were exposed to extracts *H. niger* at five different concentrations: 2, 4, 8, 16, 32 μg/mL. The processes were replicated four times for each concentration of extracts and for an untreated control group. Twenty-five larvae were used for each replicate (100 larvae were used for each concentration). Larvae were transported to a rearing plastic cup (100 cm^3^) containing a piece of beef and the test materials and were exposed to the 72 h. The plastic cups were covered with clean gauze and protected by a rubber band. In the control groups, larvae were treated using distilled water and Tween-80. Larvae were preserved under laboratory circumstances at 27 ± 2 °C, 80 ± 5% relative humidity, and a 16:8 h light:dark cycle. Larva behavior (feeding and movement activity) was observed at 8, 12, 24, 36, 48 and 72 h (until 3rd instar). Larvae mortality amounts were determined till pupation. Larvae were measured alive if they displayed normal behavior when breathed upon or physically stimulated with wooden dowels; larvae unable of movement and not maintaining any signs of life were measured moribund or dead [42,43].

### 4.8. HPLC Conditions

HPLC studies were conducted using an Agilent Technologies HP 1100 series chromatograph equipped with a gradient pump, column oven, membrane degasser, UV detector and injector. Separation was conducted using a C-18 column (150 mm × 4.6 mm I.D., 5µm). The column was maintained at 25 °C and the mobile phase flow rate was 0.8 mL/min. Solvent A contained acetonitrile, and solvent B contained 15 mM ammonia water solution. The injection volume was 10 µL. The following gradient program was used as 0–6 min: isocratic at 10% A; 6–12 min: linear gradient from 10% to 40% A; 12–20 min: isocratic at 40% A; 20–25 min: linear gradient from 40% to 85% A; 25–30 min: linear gradient from 85% to 10% A. A 5 min delay was maintained for equilibration of the column and stabilization of the baseline. Total analysis time was 35 min. The peaks were documented at 205 nm. Quantitation of the alkaloids found in plant samples used a seven-point linear regression curve for scopolamine and hyoscyamine. The calculated mean amount of alkaloid (*g/g*) was based on the weight of the ground dry plants [44].

### 4.9. Statistical Analysis

Data were statistically examined using ANOVA to test the changes among the five concentrations of *H. niger* and control means. Duncan’s test was used to separate means (*p* < 0.05) using GraphPad Prism 6.0. The percentage of mortalities caused from larvae treated using *H. niger* were corrected for natural mortality according to Abbot’s formula [45].

## 5. Conclusions

In conclusion, *H. niger* seeds alkaloid extract was significantly effective against *L. sericata*. HPLC analysis revealed that hyoscyamine and scopolamine were predominant compounds in the alkaloid extract of *H. niger*. Therefore, these results confirmed the folkloric usage of this plant. Furthermore, it could be recommended the alkaloid content of the plant could be responsible for the insecticidal activity. In further studies, we are planning to test the larvicidal effects of hyoscyamine and scopolamine, which are the main constituents of the alkaloid extract of *H. niger* on *L. sericata*.

## Figures and Tables

**Table 1:** The percentage mortality of first instar *Lucilia sericata* and number developing to pupa when exposed to each of 5 concentrations of four extracts of *Hyoscyamus niger* seeds.

Extract Type	Conc. (μg/mL)	1st Instars									Pupa Number
		1st		*p* Value	Molted to 2nd		*p* Value	Molted to 3rd		*p* Value	
		No.	M.%		No.	M.%		No.	M.%		
*n*-Hexane	2	100	5 ± 1.9 ^a^	ns	95	5.3 ± 1.1 ^a^	ns	90	5.6 ± 2.3 ^a^	ns	85
	4	100	12 ± 3.2 ^a^	ns	88	5.7 ± 1.4 ^a^	ns	83	9.6 ± 3.7 ^a^	ns	75
	8	100	13 ± 5.5 ^a^	ns	87	6.9 ± 2.4 ^a^	ns	81	12.3 ± 6.9 ^a^	ns	71
	16	100	18 ± 10.8 ^a^	ns	82	9.8 ± 2.8 ^a^	ns	74	9.5 ± 5.2 ^a^	ns	67
	32	100	24 ± 16.5 ^b^	0.038	76	11.8 ± 3.7 ^a^	ns	67	10.4 ± 3.1 ^a^	ns	60
Ethyl acetate	2	100	2 ± 1.1 ^a^	ns	98	5.1 ± 2.7 ^a^	ns	93	3.2 ± 1.7 ^a^	ns	90
	4	100	7 ± 4.3 ^a^	ns	93	7.5 ± 2.9 ^a^	ns	86	5.8 ± 2.8 ^a^	ns	81
	8	100	14 ± 9.6 ^a^	ns	86	8.1 ± 3.1 ^a^	ns	79	11.4 ± 4.5 ^a^	ns	70
	16	100	19 ± 11.3 ^a^	ns	81	12.3 ± 4.2 ^a^	ns	71	12.7 ± 3.6 ^a^	ns	62
	32	100	21 ± 13.2 ^a,b^	0.049	79	15.2 ± 3.6 ^a^	ns	67	22.4 ± 9.4 ^b^	0.037	52
Methanol	2	100	12 ± 4.6 ^a^	ns	88	13.6 ± 4.7 ^a^	ns	76	19.7 ± 8.7 ^a^	ns	61
	4	100	17 ± 7.5 ^a^	ns	83	18.1 ± 6.3 ^a^	ns	68	20.6 ± 7.3 ^a^	ns	54
	8	100	22 ± 9.9 ^b^	0.042	78	20.5 ± 7.8 ^a,b^	0.047	62	27.4 ± 8.1 ^b^	0.044	45
	16	100	25 ± 9.2 ^b^	0.037	75	26.7 ± 6.1 ^b,c^	0.034	55	32.7 ± 10.5 ^c^	0.005	37
	32	100	33 ± 10.6 ^b^	0.021	67	100.0 ± 0.0 ^e^	0.000	0	0.0 ± 0.0 ^a^	ns	0
Alkaloid extract	2	100	26 ± 9.8 ^b^	0.028	74	81.1 ± 13.5 ^d^	0.019	14	100.0 ± 0.0 ^e^	0.000	0
	4	100	38 ± 8.5 ^b^	0.018	62	88.7 ± 11.4 ^d^	0.014	7	100.0 ± 0.0 ^e^	0.000	0
	8	100	60 ± 10.4 ^c^	0.011	40	100.0 ± 0.0 ^e^	0.000	0	0.0 ^a^	-	0
	16	100	81 ± 14.3 ^d^	0.000	19	100.0 ± 0.0 ^e^	0.000	0	0.0 ^a^	-	0
	32	100	100 ± 0.0 ^e^	0.000	0	100.0 ± 0.0 ^e^	0.000	0	0.0 ^a^	-	0
Control		100	1 ± 0.0 ^a^	ns	99	3.0 ± 1.6 ^a^	ns	96	0.0 ^a^	ns	96

^a,b,c,d,e^ explain the significant difference between the percent of mortalities; M—Mortality; ns—non significant.

**Table 2:** The percentage mortality of second instars *Lucilia sericata* and number developing to pupa when exposed to each of five concentrations of four extracts of *Hyoscyamus niger* seeds.

Extract Type	Conc. (μg/mL)	2nd Instars						Pupa Number
		2nd		*p* Value	Molted to 3rd		*p* Value	
		No.	M.%		No.	M.%		
*n*-Hexane	2	100	4 ± 1.9 ^a^	ns	96	4.2 ± 2.7 ^a^	ns	92
	4	100	7 ± 2.3 ^a^	ns	93	9.7 ± 3.1 ^a^	ns	84
	8	100	10 ± 2.1 ^a^	ns	90	12.2 ± 2.3 ^a^	ns	79
	16	100	13 ± 5.2 ^a^	ns	87	14.9 ± 5.4 ^a^	ns	74
	32	100	16 ± 4.9 ^a^	ns	84	22.6 ± 8.6 ^b^	0.028	65
Ethyl acetate	2	100	12 ± 2.6 ^a^	ns	88	17.0 ± 9.2 ^a^	ns	73
	4	100	15 ± 6.2 ^a^	ns	85	20.0 ± 12.9 ^a,b^	0.039	68
	8	100	21 ± 8.3 ^a,b^	0.035	79	22.7 ± 10.8 ^b^	0.020	61
	16	100	25 ± 7.5 ^b^	0.014	75	32.0 ± 18.2 ^b^	0.008	51
	32	100	32 ± 9.8 ^b^	0.020	68	41.2 ± 15.3 ^b,c^	0.000	40
Methanol	2	100	22 ± 9.1 ^a,b^	0.024	78	23.1 ± 10.4 ^b^	0.018	60
	4	100	27 ± 6.4 ^b^	0.007	73	32.9 ± 17.1 ^b^	0.000	49
	8	100	34 ± 8.0 ^b^	0.003	66	45.5 ± 14.8 ^c^	0.000	36
	16	100	45 ± 11.6 ^c^	0.000	55	47.3 ± 18.9 ^c^	0.000	29
	32	100	52 ± 19.3 ^c^	0.000	48	52.1 ± 17.3 ^c^	0.000	23
Alkaloid extract	2	100	25 ± 8.5 ^b^	0.017	75	25.3 ± 10.8 ^b^	0.011	56
	4	100	38 ± 10.9 ^b^	0.000	62	51.6 ± 13.4 ^c^	0.000	30
	8	100	47 ± 9.3 ^c^	0.000	53	75.5 ± 9.6 ^d^	0.000	13
	16	100	89 ± 11.2 ^d^	0.000	11	0.0 ± 0.0 ^a^	-	0
	32	100	100 ± 0.0 ^e^	0.000	0	0.0 ± 0.0 ^a^	-	0
Control		100	0 ± 0.0 ^a^	ns	100	2.0 ± 0.6 ^a^	ns	98

^a,b,c,d,e^ explain the significant difference between the percent of mortalities; M—Mortality; ns—non significant.

**Table 3:** The percentage mortality of third instars *Lucilia sericata* and number developing to pupa when exposed to each of five concentrations of four extracts of *Hyoscyamus niger* seeds.

Extract Type	Conc. (μg/mL)	3rd Instars		*p* Value	Pupa Number
		No.	M.%		
*n*-Hexane	2	100	3 ± 1.8 ^a^	ns	97
	4	100	12 ± 6.4 ^a^	ns	88
	8	100	14 ± 4.9 ^a^	ns	86
	16	100	20 ± 12.3 ^a^	ns	80
	32	100	23 ± 11.8 ^a,b^	0.045	77
Ethyl acetate	2	100	18 ± 7.3 ^a^	ns	82
	4	100	25 ± 9.6 ^b^	0.031	75
	8	100	28 ± 8.0 ^b^	0.024	72
	16	100	32 ± 10.4 ^b^	0.011	68
	32	100	41 ± 13.7 ^c^	0.000	59
Methanol	2	100	17 ± 18.2 ^a^	ns	73
	4	100	36 ± 15.1 ^b^	0.003	64
	8	100	48 ± 19.7 ^c^	0.000	52
	16	100	54 ± 16.5 ^c^	0.000	46
	32	100	62 ± 27.4 ^c^	0.000	38
Alkaloid extract	2	100	29 ± 5.6 ^b^	0.020	71
	4	100	40 ± 9.5 ^c^	0.000	60
	8	100	69 ± 10.7 ^d^	0.000	31
	16	100	100 ± 0.0 ^e^	0.000	0
	32	100	100 ± 0.0 ^e^	0.000	0
Control		100	0 ± 0.0 ^a^	ns	100

^a,b,c,d,e^ explain the significant difference between the percent of mortalities; M—Mortality; ns—non significant.
